# Tuning the electrical transport of type II Weyl semimetal WTe_2_ nanodevices by Ga+ ion implantation

**DOI:** 10.1038/s41598-017-12865-8

**Published:** 2017-10-04

**Authors:** Dongzhi Fu, Bingwen Zhang, Xingchen Pan, Fucong Fei, Yongda Chen, Ming Gao, Shuyi Wu, Jian He, Zhanbin Bai, Yiming Pan, Qinfang Zhang, Xuefeng Wang, Xinglong Wu, Fengqi Song

**Affiliations:** 1 National Laboratory of Solid State Microstructures, Collaborative Innovation Center of Advanced Microstructures, and College of Physics, Nanjing University, Nanjing, 210093 P. R. China; 2 National Laboratory of Solid State Microstructures, Collaborative Innovation Center of Advanced Microstructures, and School of Electronic Science and Engineering, Nanjing University, Nanjing, 210093 P. R. China; 3 Key Laboratory for Advanced Technology in Environmental Protection of Jiangsu Province, Yancheng Institute of Technology, Yancheng, 224051 P. R. China

## Abstract

Here we introduce lattice defects in WTe_2_ by Ga+ implantation (GI), and study the effects of defects on the transport properties and electronic structures of the samples. Theoretical calculation shows that Te Frenkel defects is the dominant defect type, and Raman characterization results agree with this. Electrical transport measurements show that, after GI, significant changes are observed in magnetoresistance and Hall resistance. The classical two-band model analysis shows that both electron and hole concentration are significantly reduced. According to the calculated results, ion implantation leads to significant changes in the band structure and the Fermi surface of the WTe_2_. Our results indicate that defect engineering is an effective route of controlling the electronic properties of WTe_2_ devices.

## Introduction

Since the discovery of graphene^1–3^ and topological insulators^4–7^, the study of various new topological materials has become a hot spot. Recent years Weyl semimetals (WSMs), first implemented in the TaAs family^8–11^, have aroused a wide range of interest due to their unique band structures and transport properties. Many prediction including Weyl point, Weyl cone, Fermi arc and chirality anomaly have been demonstrated by the experiments including angle-resolved photoemission spectroscopy^9,10,12–14^, negative longitudinal magnetoresistance^15–17^. Recently, a type of WSM, type II WSM, is proposed and verified in WTe_2_^18–22^, MoTe_2_^23–27^ and Mo_x_W_1−x_Te_2_^28–30^, etc. It is characterized by a few sets of heavily titled Weyl cones, whose Weyl points appear at the contact of electron and hole pockets. Due to the unique properties of the band, the theory predicts that there are many unique properties different from those of type I: momentum space Klein tunneling^31^, field-selective chiral anomaly^32^ and intrinsic anomalous Hall Effect^33^, which becomes a good platform with intense research efforts.

For WTe_2_, an extremely large positive magnetoresistance (XMR) has been observed, which has a value of 13,000,000% up to 60T at 0.53K with current along a- axis and magnetic field parallel to c-axis^34^. Monolayer WTe_2_ with 1T′structure is predicted theoretically to be quantum spin Hall insulator^35^ and edge conduction, which is confirmed recently^36^. Pressure-induced dome-shaped superconductivity is reported in WTe_2_ with a maximum critical temperature (Tc) of 7 K at around 16.8 GPa^37,38^. Recently WTe_2_ is predicted to be type II WSM^18^ and chiral anomaly induced negative magnetoresistance is observed in WTe_2_ devices of thin film and bulk materials^39–41^. However, theoretical and experimental studies on lattice defects and their kinetics of WTe_2_ are still absent. Lattice defects have a significant impact on the properties of WTe_2_, so research on this is urgently needed. As the first theoretical candidate of type II WSM, WTe_2_ have four pairs of Weyl points which are believed to be 50–60meV higher than the Fermi level of the pristine solid^18^. Such a subtle control can often be tackled by the defect engineering^42,45^.

Ion implantation is an important way to introduce lattice defects in crystals, and has been widely used in the past research^42–45^. Here we report that, GI is carried out on WTe_2_ nanodevices of thin flakes. Raman characterization, transport measurement and theoretical calculation determine the dominant defects and its effect on the device transport.

## Results and Discussion

**Figure 1:**
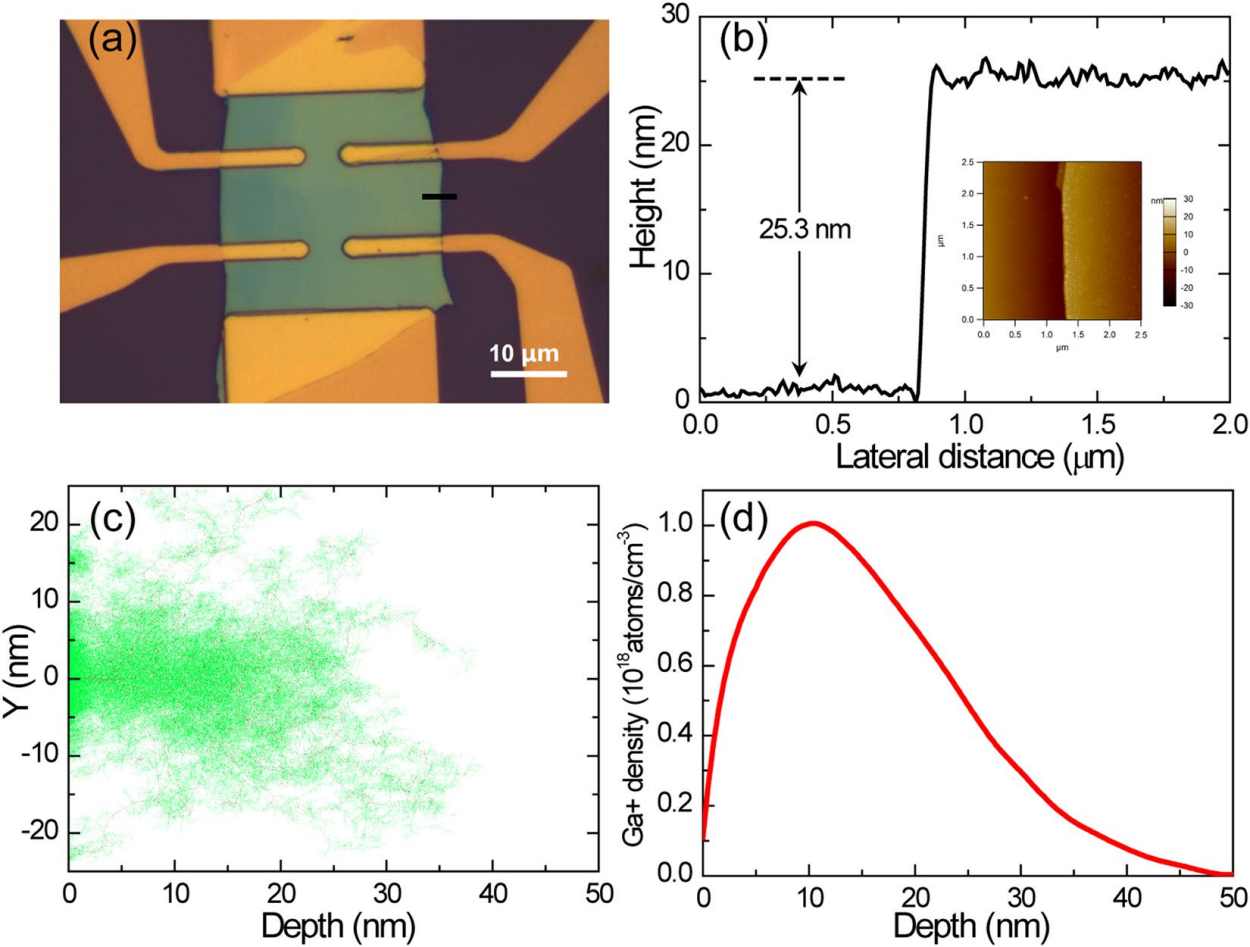
Device configuration and SRIM simulation for GI. (**a**) Typical optical image of our WTe_2_ thin film devices with Hall-bar geometry. (**b**) Atomic force microscope (AFM) height profile of the flake along the black line. Inset shows the original image of atomic force microscopy along the black line. (**c**) Simulation for 100 Ga+ implantation into WTe_2_ flakes. The simulated diagram was got by software “The Stopping and Range of Ion in Matter” (SRIM) with ion energy 30 keV. The red dots show the vacancies created by Ga+, while the green dots are vacancies caused by recoiling W or Te atoms. (**d**) Ga+ density profile of the WTe_2_ film with GI. Ten thousand Ga+ with ion energy 30keV were used in SRIM to obtain the distribution curve.

WTe_2_ is a member of laminar transition metal dichalcogenides (TMDs). Unlike other TMDs, the Td phase is the naturally stable structure of WTe_2_. Due to the weak Van der Waals force between different layers, WTe_2_ can be easily exfoliated into films with atomic thickness. In our work, WTe_2_ thin flakes are obtained by mechanical cleavage of strip-like crystals onto 285 nm-SiO_2_/Si substrates. Figure 1a shows a typical optical image of the WTe_2_ devices with Hall-bar geometry, and the thickness of the thin flake is ~25.3 nm, determined from the height profile of the film along the black line plotted in Fig. 1b. Inset of Fig. 1b shows original image of atomic force microscopy along the black line.

Here ion implantation is carried out in order to introduce structural defects in the 3D semimetal WTe_2_. GI is carried out in a FEI Helios Nanolab 600i dual beam system with Ga+ ion sources. A detailed description of ion implantation process is given in the method section. The candidate structural defects created by ion implantation include Frenkel defects and substitution defects, whose influence on transport properties and band structure will be elaborated in the following section. Here the software “The Stopping and Range of Ion in Matter” (SRIM) is used to simulate the process of GI. Figure 1c show the simulation for 100 Ga+ with ion energy 30 keV implantation into WTe_2_ flake. The ion-induced vacancies (strike a W or Te atom away from its lattice site) are represented by red dots, and the vacancies caused by the recoiling W/Te atoms are indicated by green dots. Easy to find that the ions cause lattice damage (red dots) constantly, while recoiling W/Te atoms always create recoil cascade shown by clusters of green dots. Ga+ density profile of the WTe_2_ film derived from SRIM simulation was shown in Fig. 1d with operating voltage 30kV. Known from the curve, by means of GI we can get a relatively uniform concentration distribution of Ga+ ion in WTe_2_ thin film samples with thickness of ~25 nm. The detailed dependence of energy and projected range is shown in Table S2 of the supplementary information.

**Figure 2:**
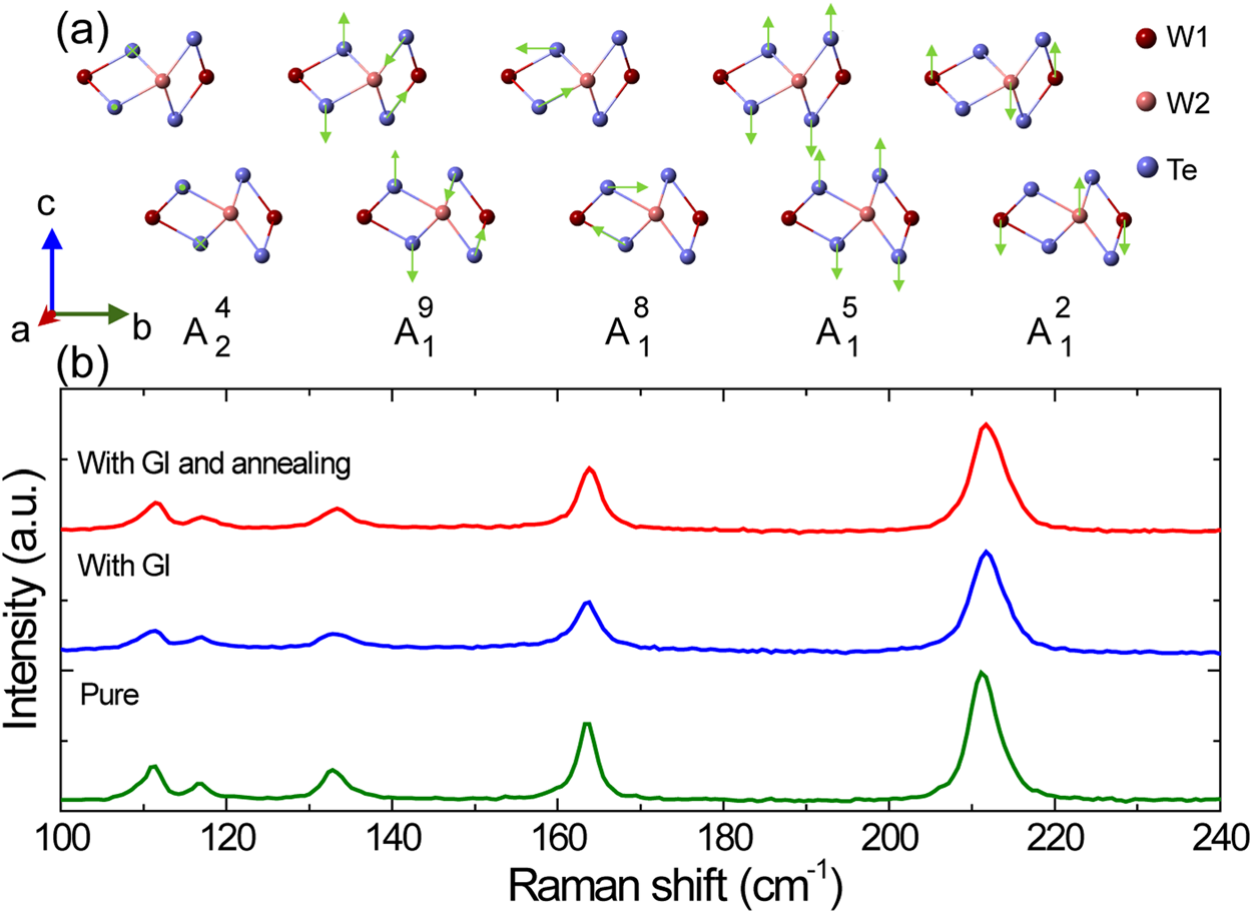
Raman evidence for main defects in WTe_2_ induced by GI. (**a**) Atomic displacements of Raman active modes in WTe_2_. Here, “×” and “·” indicate that the Te atoms move into and out of the *bc* plane, respectively. (**b**) Raman spectra of WTe_2_ flakes with incident laser along the c-axes at room temperature. The green, blue and red line show the Raman spectra for pure sample A, sample A with GI and sample A with GI and annealing, respectively.

In order to determine the dominant type of structure defects, we characterize our samples by Raman spectroscopy. The calculated results about all 33 Raman vibrational modes in ref.^46^ are applied in our study. Figure 2a show all the five Raman vibrational modes observed in sample A (~8.5 nm) with laser applied in the $z(\mathrm{xx})\bar{{\rm{z}}}$ geometry. As shown in Fig. 2a, the first four modes (${{\rm{A}}}_{2}^{4}$, ${{\rm{A}}}_{1}^{9}$, ${{\rm{A}}}_{1}^{8}$ and ${{\rm{A}}}_{1}^{5}$) are induced by the relative movements of Te atoms, while the ${{\rm{A}}}_{1}^{2}$ mode is caused by the displacement between neighbouring W1 and W2 atoms^46^. Among all the five modes, pure longitudinal and transverse optical modes are represented by ${{\rm{A}}}_{2}^{4}$ and ${{\rm{A}}}_{1}^{5}$ respectively^46^.

Raman spectra of sample A with incident Laser along the c-axes at room temperature is shown in Fig. 2b. The green line show the experimental data for pure sample A (without GI and annealing), blue line represent the Raman spectra for sample A with GI (irradiated to total doses of 0.44 μC. cm^−2^) and red line is the Raman spectra for sample A with GI and annealing. All the peak appearing in the range from 100~250 cm are analyzed by Lorentz fitting, and the results are shown in Table 1. On the basis of calculation results in ref. ^46^,five Raman vibrational modes are identified as shown in Fig. 2b. The ${{\rm{A}}}_{2}^{4}(\sim 111.1{{\rm{cm}}}^{-1})$, ${{\rm{A}}}_{1}^{9}(\sim 116.7{{\rm{cm}}}^{-1})$, ${{\rm{A}}}_{1}^{8}(\sim 133{{\rm{cm}}}^{-1})$, ${{\rm{A}}}_{1}^{5}(\sim 163.5{{\rm{cm}}}^{-1})$, and ${{\rm{A}}}_{1}^{2}(\sim 211.4{{\rm{cm}}}^{-1})$ modes were allotted in sequence for pure sample A, which agrees with the results in ref.^46^. In the meantime, the ${{\rm{A}}}_{2}^{4}(\sim 111{{\rm{cm}}}^{-1})$, ${{\rm{A}}}_{1}^{9}(\sim 116.8{{\rm{cm}}}^{-1})$, ${{\rm{A}}}_{1}^{8}(\sim 133.2{{\rm{cm}}}^{-1})$, ${{\rm{A}}}_{1}^{5}(\sim 163.5{{\rm{cm}}}^{-1})$, and ${{\rm{A}}}_{1}^{2}(\sim 211.8{{\rm{cm}}}^{-1})$ modes were allotted sequentially for sample A with GI. The${{\rm{A}}}_{2}^{4}(\sim 111.3{{\rm{cm}}}^{-1})$, ${{\rm{A}}}_{1}^{9}(\sim 117.1{{\rm{cm}}}^{-1})$, ${{\rm{A}}}_{1}^{8}(\sim 133.3{{\rm{cm}}}^{-1})$, ${{\rm{A}}}_{1}^{5}(\sim 163.8{{\rm{cm}}}^{-1})$, and ${{\rm{A}}}_{1}^{2}(\sim 212{{\rm{cm}}}^{-1})$ modes were allotted sequentially for sample A with GI and annealing.

**Table 1:** Raman parameters comparison for pure sample A, sample A with GI and sample A with GI and annealing.

Sample A	Mode	Peak position/cm^−1^			Full width at half maximum/cm^−1^			Relative intensity I(Pi)/I(P5)		
		Pure	With GI	With GI and annealing	Pure	With GI	With GI and annealing	Pure	With GI	With GI and annealing
P1	${{\rm{A}}}_{2}^{4}$	111.1	111	111.3	2.95	3.8	3.55	0.25795	0.18738	0.24337
P2	${{\rm{A}}}_{1}^{9}$	116.7	116.8	117.1	2.6	3.17	3.97	0.11159	0.09701	0.1027
P3	${{\rm{A}}}_{1}^{8}$	133	133.2	133.3	3.31	4.49	4.15	0.22329	0.14206	0.17697
P4	${{\rm{A}}}_{1}^{5}$	163.5	163.5	163.8	2.62	3.65	3.18	0.59845	0.4483	0.56087
P5	${{\rm{A}}}_{1}^{2}$	211.4	211.8	212	4.02	4.66	4.68	1	1	1

All the parameters in the table are obtained by Lorentz fitting.

First of all, the peak positions almost show no change after GI and annealing. The weak changes in peak position are comparable with measuring error of the instrument. Furthermore, the full width at half maximum (FWHM) of the Raman peaks change significantly. GI increase the FWHM by introducing lattice defects, while annealing decrease the FWHM by repairing part of the defects and improve the quality of the samples. So the significant changes of the FWHM have confirmed the effect of “GI” and “annealing after GI” very well, which is consistent with the our expectation. Considering that no excess defect peak is observed, no major peak disappears, the peak position remain almost unchanged, we believe that the main features of pure WTe_2_ are retained in sample A with GI and annealing.

Relative intensity changes in Raman peaks provide some important clues about defect type in sample A with GI. For comparison purposes, the intensity of all Raman peaks are normalized by intensity of ${{\rm{A}}}_{1}^{2}$ modes as shown in Table 1. All the Raman vibrational modes originating from the relative movements of Te atoms weaken dramatically after GI relative to ${{\rm{A}}}_{1}^{2}$. Similar results from sample C are shown in Fig. S2 and Table S3 of the supplementary information. However ${{\rm{A}}}_{1}^{2}$ is caused by the displacement between adjacent W1 and W2 atoms. So Te defects are dominant defects relative to W defects in sample A with GI, which is consistent with the crystal structure of WTe_2_. WTe_2_ is layered material with W atomic layers sandwiched by Te atomic layers. In addition to W atom being heavier than the Te atom, each W atom forms chemical bonds with six Te atoms and each Te atom shares chemical bonds with only three W atoms. All of this will lead to that Te defects are easier to form than W defects, which has be confirmed by computational formation energy of candidate defects in the following section.

We have calculated the formation energy of possible defect types to further determine the dominant defect introduced by ion implantation in the WTe_2_ device. We divide the main defect types into two categories: Frenkel defects of Te/W atom (a vacancy plus an adjacent interstitial atom) and substitution defects (a Te/W atom replaced by a Ga atom plus a Te/W interstitial atom). The calculated results are: 0 eV for pure WTe_2_, −4.674 eV for Te vacancy and Te interstitial, −5.310 eV for W vacancy and W interstitial, −0.593 eV for Ga in Te site and Te interstitial, −2.140 eV for Ga in W site and W interstitial. In order to facilitate the comparison, the formation energy of pure WTe_2_ system can be set to 0. Here the greater the formation energy of a defect is, the more easily the formation of such kind of defect becomes. Comparing the formation energy of different defects, we can find that the formation energy of substitution defects can be significantly larger than that of Frenkel defect. In the meantime, the formation energy of Te defects can be larger than that of W defects of the same type. Considering that during the ion implantation process one implanted Ga+ ion can produce a large number of W/Te Frenkel defects and only one substitution defects, we suggest that the concentration of substitution defects is much smaller than that of the W/Te Frenkel defects. Therefore, Te Frenkel defect is the dominated defect in the WTe_2_ device with GI, which is consistent with the Raman results.

According to previous reports, WTe_2_ is semimetal with near-perfect electron-hole compensation^34,47,48^, and its transport behavior can be expressed using the following formula^49,50^:

1 $${\rho }_{{\rm{xx}}}=\frac{(n{\mu }_{{\rm{e}}}+p{\mu }_{{\rm{h}}})+(n{\mu }_{{\rm{e}}}{\mu }_{{\rm{h}}}^{2}+p{\mu }_{{\rm{e}}}^{{\rm{2}}}{\mu }_{{\rm{h}}}){B}^{2}}{e[{(n{\mu }_{{\rm{e}}}+p{\mu }_{{\rm{h}}})}^{2}+{(p-n)}^{2}{\mu }_{{\rm{e}}}^{{\rm{2}}}{\mu }_{{\rm{h}}}^{2}{B}^{2}]}$$

2 $${\rho }_{{\rm{xy}}}=\frac{(p{\mu }_{{\rm{h}}}^{{\rm{2}}}-n{\mu }_{{\rm{e}}}^{{\rm{2}}})B+{\mu }_{{\rm{e}}}^{{\rm{2}}}{\mu }_{{\rm{h}}}^{2}(p-n){B}^{3}}{e[{(n{\mu }_{{\rm{e}}}+p{\mu }_{{\rm{h}}})}^{2}+{(p-n)}^{2}{\mu }_{{\rm{e}}}^{{\rm{2}}}{\mu }_{{\rm{h}}}^{2}{B}^{2}]}$$

where *n*(*p*) and μ_e_(*μ*_h_) are the carrier density and mobility for electrons (holes), respectively, and *B* is the magnetic field. Considering that both the quantum effect and disorders created by GI have a huge impact on the magnetoresistance, here we use equation (2) for two band model fit. At the same time, the following formula is used as a limiting condition:

3 $${\rho }_{{\rm{xx}}}(B=0)=\frac{1}{e(n{\mu }_{{\rm{e}}}+p{\mu }_{{\rm{h}}})}$$

All the transport parameters extracted from the two band model fit are summarized in Table 2.

**Table 2:** Parameters used for GI and that derived from the two-band model fitting.

Sample	Thickness (nm)	Operating voltage (kV)	Ga+ ions beam current (pA)	Dose (μCcm^−2^)	*n*(10^19^cm^−3^)		*p*(10^19^cm^−3^)		*n*/*p*		*μ*_e_(cm^2^v^−1^s^−1^)		*μ*_h_(cm^2^v^−1^s^−1^)	
					Before GI	After GI	Before GI	After GI	Before GI	After GI	Before GI	After GI	Before GI	After GI
B	25.3	30	2	0.4	4.460	1.750	4.339	1.723	1.028	1.016	2161	1018	1736	994

*n*, *p*, carrier density for electron and hole respectively; *n*/*p*, the ratio of carrier density; *μ*_e_, *μ*_h_, mobility for electron and hole respectively.

**Figure 3:**
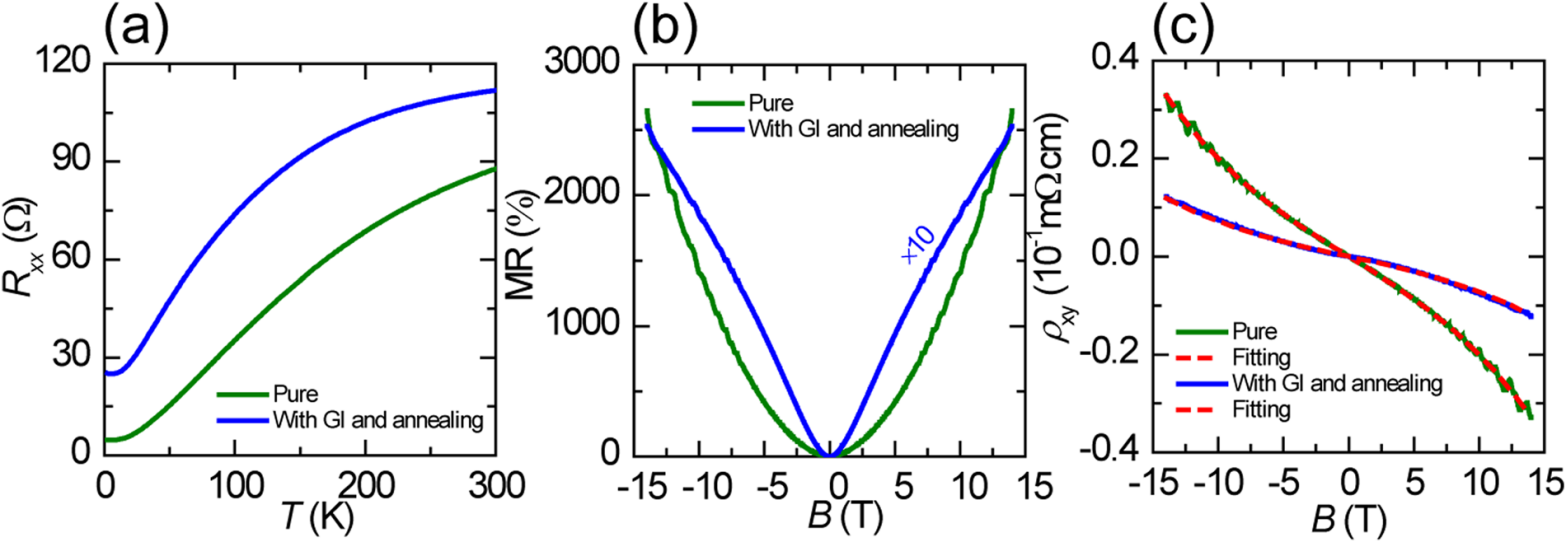
Effect of lattice defects on transport properties. (**a**) Temperature dependence of the resistance *R*_xx_ in zero field for sample B. The green line represents the experimental data for pure sample B and the blue line for sample B with GI and annealing. (**b** and **c**) display the field dependence of MR and *ρ*_xy_ at 2 K, respectively, with a magnetic field applied along the c-axis direction. The red dotted line in (**c**) represents the two band model fit for sample B.

Figure 3a shows the resistance of the sample B (~25.3 nm) as a function of temperature at *H* = 0. The green line represents the experimental data for sample B without GI and the blue line with GI and annealing. The resistance of the virgin sample shows the metallic temperature dependence expected for carriers compensated semimetal WTe_2_, i.e., *R*_xx_ decreases monotonically with decreasing temperature. For the samples B irradiated to total doses of 0.4 μC  · cm^−2^, *R*_xx_ still displays metallic temperature dependence, however the absolute values of the resistance are increased over the entire temperature range, which is due to the decrease of carriers density and mobility proved by two-band fitting as shown in Table 2.

Figure 3b depicts the magnetoresistance as the function of magnetic field *B* applied along the *c-*axes, for the virgin and irradiated sample B at 2 K. The MR is defined by MR = [*R*(*B*) − *R*(0)]/*R*(0) × 100%. At 2 K and in 14 T, the MR of virgin sample B reaches as high as 2600%, whereas GI reduces the XMR effect by an order of magnitude. The reduction in magnetoresistance is due to the decrease of mobility, which can be induced by a significant increase in native charged scattering centers. It’s very distinct that the MR of sample B change from quadratic field dependence to V-shaped over the entire field range after GI, suggesting the presence of disorder in the crystal. The disorder can be clusters of interstitial atoms and vacancies, which were observed in proton-irradiated Bi_2_Te_3_^51^. The oscillatory component superimposed onto the smoothly background corresponds to the Shubnikov-de Haas oscillation, which is very obvious in virgin sample B but weak in irradiated sample B. In general, the decay of quantum oscillation originates from the reduction in mobility of electron and hole.

The magnetic-field dependence of *ρ*_xy_ is shown in Fig. 3c for the pristine and irradiated sample B. Hall resistivity *ρ*_xy_ exhibits a nonlinear behavior (linear at low field, proportional to*B* ^3^ at high fields), which is often observed in material with multiple carriers. The significant change in *ρ*_xy_(*B*) implies that the carrier density in WTe_2_ thin flake was effectively modulated by introduction of structure defects. Similar results from sample D is observed shown in Fig. S3 of supplementary information. Here the two band model is used to reproduce correctly the hall resistivity of sample B. Table 2 summarizes the evolution of the electron and hole concentration, mobility and carriers concentration ratio, respectively. The mobility for electron decline greatly from 2161 cm^2^V^−1^s^−1^ to 1018 cm^2^V^−1^s^−1^, and the mobility for hole decay from1736 cm^2^V^−1^s^−1^ to 994 cm^2^V^−1^s^−1^, which stem from the emergence of a large number of scattering centers. As shown in the Table 2, after GI, both electron and hole densities decrease obviously, implying the change of band structure and Fermi level in WTe_2_ flake. Interestingly, carrier concentration ratio is almost unchanged in sample B with GI, suggesting that electron-hole compensation is maintained. However sample D with thinner thickness exhibits breakdown of electron-hole compensation shown in Table S4 (supplementary information). So introduction of structure defects by ion implantation is an effective method to modulate the carrier density, especially in semimetal devices, which cannot be tuned effectively by traditional gating due to the limit of screen length.

**Figure 4:**
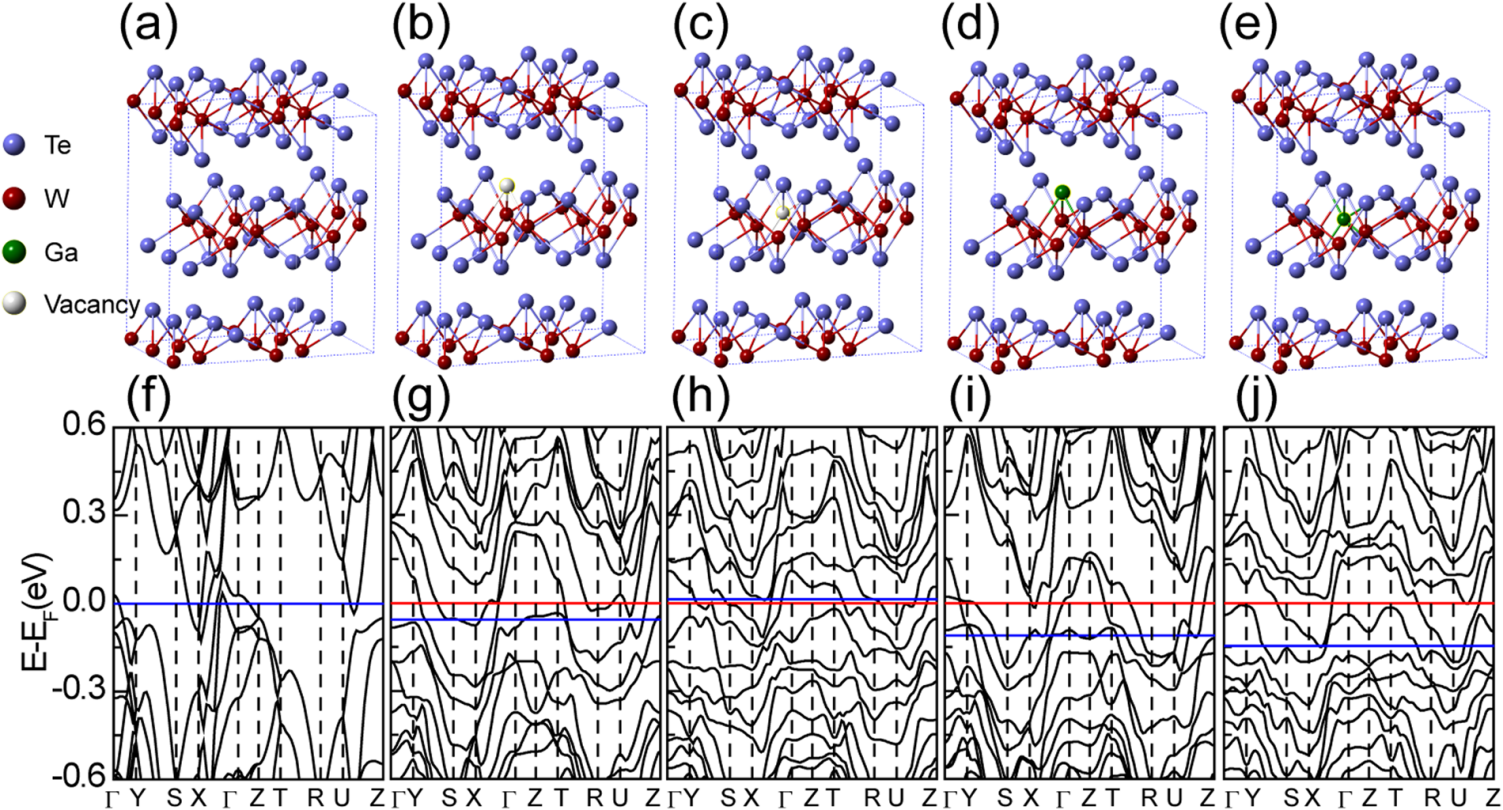
Band structure of WTe_2_ with various lattice defects. (**a**) Crystal structure of WTe_2_ with a tungsten chain along the a axis. Blue and dark red spheres represent Te and W, respectively. (**b**–**e**) Structure diagrams for different types of lattice defects in WTe_2_ flakes induced by ion implantation, the interstitial Te/W not shown in the figures. Te/W vacancies and Ga atoms are shown by spheres with color white and green respectively. (**f**) Band structure of WTe_2_. (**g**–**j**) Band structure of WTe_2_ with a certain concentration of defects as (**b**–**e**), respectively. The blue line in (**h**) is moved upwards to be seen clearly.

In addition to Raman characterization and transport measurements, we also investigate the impact of GI on the band structure and Fermi surface of WTe_2_. We performed our calculation by the Vienna Ab-initio Simulation Package (VASP) code^52–55^. The distribution of high symmetry points in the first Brillouin zone is shown in Fig. S4 in supplementary information. Figure 4a–e are the crystal structure diagrams of pure WTe_2_ and all of the defect types we consider in this work, including Te/W atom Frenkel defects and substitution defects. At the same time, Fig. 4f–j are the corresponding band structure for pure WTe_2_ and WTe_2_ crystal with a certain concentration of specific defects. As shown in Fig. 4f–j, the red line indicates the current position of Fermi energy for WTe_2_ crystal with a certain concentration of specific defects, while the blue line represents the position of the Fermi energy for the pure WTe_2_ crystal. As can be seen from Fig. 4f–j, the introduction of a certain concentration of structure defects in pure WTe_2_ crystal, will result in a significant change in the band structure. In addition, the location of the Fermi surface will be significantly changed. The introduction of a certain concentration of W atom Frenkel defect has little effect on the Fermi energy of the WTe_2_ crystal, while the introduction of the remaining three defects leads to a significant lifting in Fermi energy. The changes in Fermi level caused by various crystal defects are recorded in Table S5 in supplementary information. Considering that GI can cause a decrease in carrier concentration, we can speculate that Te atom Frenkel defect as the main defect type, is likely to result in the reduction of density of state (DOS). However the effect of other types of defects on the DOS remains to be studied.

## Conclusion

In summary, by GI, we have effectively introduced lattice defects in WTe_2_ thin film devices. Through Raman analysis and formation energy calculation, Te Frenkel defect is identified as the dominant defects in WTe_2_. Transport measurements help us analyze the effects of lattice defects. In particular, the two band model analysis shows a significant decrease in electron and hole concentration induced by Te Frenkel defects. By theoretical calculation, we know the effect of all the candidate defects on band structure and Fermi surface. Ion implantation is expected to become a new way to control the electronic properties for WTe_2_ nanodevices, meantime this approach may also apply to thin film devices of other layered TMDs.

## Methods

### Devices fabrication

WTe_2_ thin flakes were obtained by mechanically exfoliating bulk single crystals onto a Si wafer covered with a 285-nm thick thermally grown SiO_2_ layer. Optical microscopy and atomic force microscopy were used to characterize the quality and thickness of the thin flakes. Once thin flakes were identified on the substrate, conventional nano-fabrication techniques (photolithography, standard electron beam evaporation and lift off) were employed to attach electrical contacts (consisting of Ti/Au bilayers, typically 5/50 nm thick).

### Ga+ ion implantation

GI were carried out in a FEI Helios Nanolab 600i dual beam system with Ga+ ion sources at room temperature. The size of the Ga+ ion beam spot was set to 25 um × 20 um which can cover all the channel areas of WTe_2_ devices, with uniform beam current 2pA. Working voltage of dual beam system was set to 30kV which corresponds to a projection range of 13.7 nm. The duration of implantation was limited to 3s to avoid the serious degradation of transport property of WTe_2_ devices.

### Annealing

After the process of GI, annealing treatment was carried out on the devices to repair the most cascade damage originating from the ion collision. WTe_2_ devices were annealed in a horizontal tube furnace equipped with a 1 inch diameter quartz tube. Argon(89%)/H2(11%)/mixed gases were kept at a flow rate of 100 sccm with mechanical vacuum pump working. The tube furnace was heated from room temperature to 240 °C within 60 min. After maintaining at 240 °C for 120 min, the system was naturally cooled down to room temperature.

### Raman characterization

Raman spectra were characterized from a micro-Raman spectrometer (LabRAM HR) with a 514.5 nm wavelength Ar+ laser as the excitation source. The laser spot size on samples was a few microns in diameter, and the incident laser power was below 1 mW, which did not damage the studied devices.

### Transport measurements

Four-probe measurements of magnetoresistance and Hall resistivity were conducted in a commercial cryostat (Quantum Design PPMS-14).

### Electronic structure calculation

We performed our calculation by the Vienna Ab-initio Simulation Package (VASP) code^52–55^. The Kohn-Sham equation have been solved variationally in a plane wave basis set using the projector-augmented-wave (PAW) method. The exchange correlation energy was described by the functional of Perdew, Burke, and Ernzerhof (PBE)^53,56^ based on the generalized gradient approximation.

## Electronic supplementary material

Tuning the electrical transport of type II Weyl semimetal WTe2 nanodevices by Ga+ ion implantation
