# Supplementary material for: Tuning the electrical transport of type II Weyl semimetal WTe2 nanodevices by Ga+ ion implantation
Source: Sci Rep. 2017 Oct 4;7:12688. doi: 10.1038/s41598-017-12865-8 (PMC5627286; doi:10.1038/s41598-017-12865-8)
Supplement: Supplementary file 1 — Tuning the electrical transport of type II Weyl semimetal WTe2 nanodevices by Ga+ ion implantation [file 41598_2017_12865_MOESM1_ESM.pdf]

Supplementary Information

Tuning the electrical transport of type II Weyl semimetal WTe_2 nanodevices by Ga+ ion implantation

Dongzhi Fu[1,*], Bingwen Zhang[1,*],Xingchen Pan[1], Fucong Fei[1], Yongda Chen[2], Ming Gao[2], Shuyi Wu[1], Jian He[1], Zhanbin Bai[1], Yiming Pan[1], Qinfang Zhang[3], Xuefeng Wang[2], Xinglong Wu[1]& Fengqi Song[1]

[1] National Laboratory of Solid State Microstructures, Collaborative Innovation Center of Advanced Microstructures, and College of Physics, Nanjing University, Nanjing, 210093, P. R. China

[2]National Laboratory of Solid State Microstructures, Collaborative Innovation Center of Advanced Microstructures, and School of Electronic Science and Engineering, Nanjing University, Nanjing, 210093, P. R. China

[3]Key Laboratory for Advanced Technology in Environmental Protection of Jiangsu Province, Yancheng Institute of Technology Yancheng 224051, P. R. China

Correspondence and requests for materials should be addressed to F.S. , Q. Z. (email: songfengqi@nju.edu.cn; qfangzhang@gmail.com) , * These authors contributed equally to this work.

Firstly, we rule out the possibility of interference from samples aging or damage during GI and annealing. The AFM images of sample E under different conditions is shown in Fig. S1. Table S1 show the parameters related to thickness and the roughness of the sample`s surface.

There is oxidation on the top surface of the WTe_2 sample. But the oxidation phenomenon is limited to top surface of sample, and there are no obvious Raman peak position shifts during the degradation in both 2L and 3L WTe_2 as reported in another work[1]. The oxidation products such as WO_x and TeO_2 will passivate the WTe_2 surface and prevent oxygen from further diffusing into inside of lattice and protect inner layer of WTe_2, which has been reported in previous work[1]. The thickness of the samples we used for Raman or transport measurement in this work are from 8.5nm to 25.3nm (12-36 layers), so the effect of surface oxidation is very limited. In short, oxidation has no significant observable effect in our study.

The Ga+ ion we used for ion implantation is a single ion, and the caused damage by each ion is very limited. GI can only cause damage in the atomic level, and will not cause obvious changes in appearance and roughness as shown in Fig S1. Though some difference are observed in the parameters, the changes of thickness, root mean square and average deviation are not larger than the resolution (~100 pm) of our AFM. So we can rule out the possibility of interference from samples damage during GI and annealing.

Here, the dependence of the implantation depth and the energy is discussed. SRIM is used to acquire the energy dependent projected range shown in Table S2. Projected range is the depth of the maximum Ga+ concentration.

Figure S2 show the Raman spectra from sample C with thickness about 17.2 nm. The parameters extracted from the Raman spectra are shown in Table S3. It is obvious that all the Raman vibrational modes (except the weakest mode A_1^9) originating from the relative movements of Te atoms weaken dramatically after GI (irradiated to total doses of $0.44\mu C \cdot cm^{-2}$) relative to A_1^2. Combining the calculated results of the formation energy in the main text, the Te Frenkel defect can also be determined as the dominant defect in sample C with GI.

Figure S3 shows the transport measurement data from sample D with thickness about 15.7 nm. The inset of Fig. S3(b) show the low field part of the MR, and obvious weak anti-localization (WAL) can be observed, which suggest the presence of disorder in the crystal. Parameters used for GI and that derived from the two band-model fitting are shown in Table S4. Ga+ ion implantation lead to a significant reduction in carrier concentration and mobility. However, unlike sample B, ion implantation seems to break the carrier balance in sample D, with n/p changing from 0.941 to 0.824.

Figure S4 shows the distribution of high symmetry points in the first Brillouin zone.

The changes in Fermi level caused by various crystal defects are recorded in Table S5.

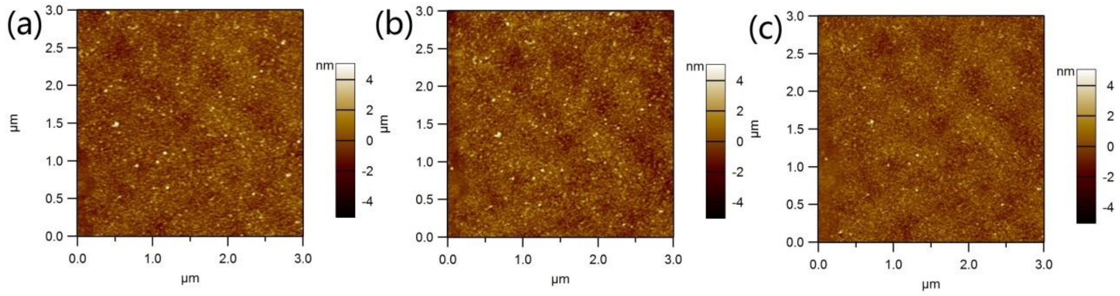

Figure S1. The AFM images of sample E, (A) pure sample E; (b) sample E with GI; (c) sample E with GI and annealing.

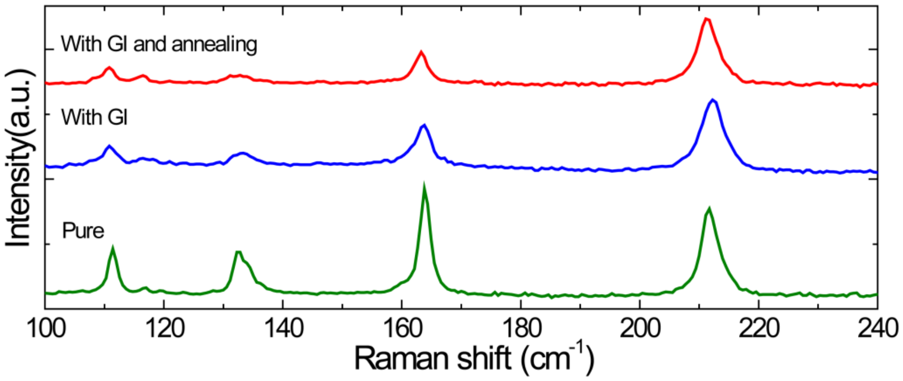

Figure S2. Raman spectra of sample C with incident laser along the c-axes at room temperature. The green, blue and red line shows the Raman spectra for pure sample C, sample C with GI and sample C with GI and annealing, respectively.

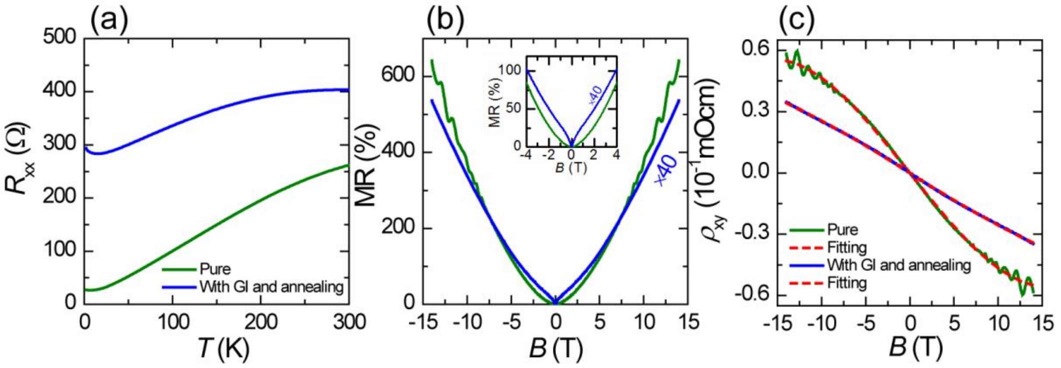

Figure S3. Effect of lattice defects on transport properties for sample D.

(a) Temperature dependence of the resistance R_{xx} in zero field for sample D. The green line represents the experimental data for pure sample D and the blue line for

sample D with GI and annealing. **(b)** and **(c)** display the field dependence of magnetoresistance ratio (MR) and ρ_{xy} at 2 K, respectively, with a magnetic field applied along the c-axis direction. The red dotted line in (c) represents the two band model fitting for sample D.

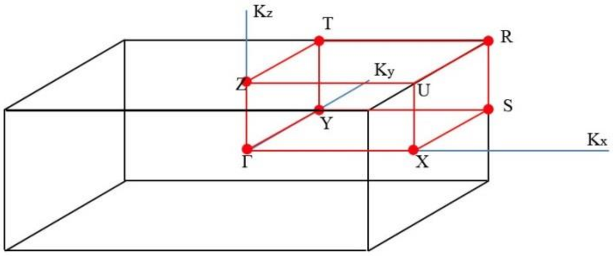

Figure S4. The distribution of high symmetry points in the first Brillouin zone.

Thickness/nm			Root mean square/pm			Average deviation/pm		
Pure sample	Sample with GI	Sample with GI and annealing	Pure sample	Sample with GI	Sample with GI and annealing	Pure sample	Sample with GI	Sample with GI and annealing
4.3	4.4	4.4	749	801	723	574	612	551

Table S1. Some parameters got by analyzing the AFM images of sample E.

Energy/eV	5	10	20	30	40	50	60	70	80
Projected range/nm	4.2	6.4	10.2	13.7	17.1	20.3	23.6	26.7	29.9

Table S2. The dependence of energy and projected range.

Sample C	Mode	Peak position/cm^{-1}			Full width at half maximum/cm^{-1}			Relative intensity I(Pi)/I(P5)		
		Pure	With GI	With GI and annealing	Pure	With GI	With GI and annealing	Pure	With GI	With GI and annealing
P1	A_2^4	111.4	111	110.8	1.68	2.52	2.37	0.516	0.238	0.216

P2	A_1^9	116.8	116.8	116.3	0.86	2.94	1.47	0.060	0.064	0.086
P3	A_1^8	133	133.2	132.6	3.34	3.88	5.18	0.491	0.170	0.121
P4	A_1^5	163.9	163.6	163.2	2.12	3.25	2.72	1.214	0.551	0.457
P5	A_1^2	211.8	212.2	211.4	3.61	4.37	3.96	1	1	1

Table S3. Raman parameters comparison for pure sample C, sample C with GI and sample C with GI and annealing. All the parameters in the table are obtained by Lorentz fitting.

Sample	Thickness (nm)	Operating voltage (kV)	Ga+ ions beam current (pA)	Dose (μCcm^{-2})	$n\left(10^{19}\,\mathrm{cm}^{-3}\right)$		$p\left(10^{19}\,\mathrm{cm}^{-3}\right)$		n/p		$\mu_e\left(\mathrm{cm}^2\mathrm{V}^{-1}\mathrm{s}^{-1}\right)$		$\mu_h\left(\mathrm{cm}^2\mathrm{V}^{-1}\mathrm{s}^{-1}\right)$	
					Before GI	After GI	Before GI	After GI	Before GI	After GI	Before GI	After GI	Before GI	After GI
D	15.7	30	1.9	0.38	2.824	1.566	3.000	1.900	0.941	0.824	2054	300	1195	235

Table S4. Parameters used for GI and that derived from the two-band model fitting. n, p, carrier density for electron and hole respectively; n/p, the ratio of carrier density; μ_e, μ_h, mobility for electron and hole respectively.

Defect types	Te vacancy and Te interstitial	W vacancy and W interstitial	Ga in Te site and Te interstitial	Ga in W site and W interstitial
Change of Fermi level (eV)	0.0569	-0.0022	0.1109	0.1464

Table S5. The changes in Fermi level caused by various crystal defects

References:

1. Ye, F. *et al.* Environmental Instability and Degradation of Single- and Few-Layer WTe2 Nanosheets in Ambient Conditions. *Small* **12**, 5802-5808, doi:10.1002/smll.201601207 (2016).
